# The gender and age perspectives of allostatic load

**DOI:** 10.3389/fmed.2024.1502940

**Published:** 2024-12-17

**Authors:** Nikola Volarić, Dunja Šojat, Mile Volarić, Ivan Včev, Tanja Keškić, Ljiljana Trtica Majnarić

**Affiliations:** ^1^Department of Pathophysiology, Physiology and Immunology, Faculty of Dental Medicine and Health, Josip Juraj Strossmayer University of Osijek, Osijek, Croatia; ^2^Department of Family Medicine, Faculty of Medicine, Josip Juraj Strossmayer University of Osijek, Osijek, Croatia; ^3^Department of Gastroenterology and Hepatology, University Clinical Hospital Mostar, Mostar, Bosnia and Herzegovina; ^4^Faculty of Medicine, Josip Juraj Strossmayer University of Osijek, Osijek, Croatia; ^5^Department of Interdisciplinary Areas, Faculty of Dental Medicine and Health, Josip Juraj Strossmayer University of Osijek, Osijek, Croatia; ^6^Department of English and German Studies, Faculty of Education, Josip Juraj Strossmayer University of Osijek, Osijek, Croatia; ^7^Department of Biomedicine, Technology and Food Safety, Laboratory of Chemistry and Microbiology, Institute for Animal Husbandry, Belgrade, Serbia

**Keywords:** age, allostatic load, chronic disease, coping behavior, gender, multimorbidity, sex, stress

## Abstract

The role of chronic stress in the development of chronic diseases, especially multimorbidity, through the pathways of increasing allostatic load, and finally, allostatic overload (the state when a compensatory mechanism is likely to fail) is being emphasized. However, allostatic load is a dynamic measure that changes depending on sex, gender, age, level and type of stress, experience of a stressful situation, and coping behaviors. Many other factors such as race, ethnicity, working environment, lifestyle, and circadian rhythm of sleep are also important. The aim of this paper was to synthesize the available information on allostatic load differences, especially those connected to sex/gender and age, and to provide a model for the future study of allostatic load, with a focus on these differences. By carefully studying allostatic load factors, we realized that many studies do not take this allostatic load difference into account in the analysis methods. In this paper, we also support the idea of further research to develop new allostatic load analysis strategies that will include all knowledge about sex/gender differences and that will, in more detail, explain numerous changeable social and educational factors that are currently accepted as biological ones. Furthermore, specific allostatic load biomarkers are expressed differently in different age groups, indicating that the discrepancies cannot be attributed solely to sex/gender disparities. This kind of approach can be valuable, not only for better explaining the differences in the frequency and age of onset of chronic diseases and multimorbidity, but also for the potential planning and development of preventive actions based on the aforementioned sex/gender and age disaparities, in order to prevent the most frequent diseases and to establish specific biomarker cut-off values for each sex/gender and age group.

## 1 Introduction

In the 1990s, McEwen and Stellar presented a theory based on allostasis — an organism’s capacity to achieve stability through change. This theory explains some of the connections between chronic stress and the onset of chronic diseases; since then, it has been the subject of many studies. Throughout their life cycle, it is commonly understood that people must modify their morphology, physiology, and behavior, and that these changes occur as everyday routines. The foundation of these modifications is the traditional idea of homeostasis (1, 2). In the literature, the term allostasis is equated with the term reactive homeostasis (response to unexpected events), thus distinguishing it from the term predictive homeostasis in a narrower sense, representing predictable circadian changes (3).

Research on the stress-disease cascade has been further developed using the allostatic load (AL) model. This model provides a comprehensive theoretical framework for measuring and conceptualizing long-term stress, considering the brain’s role in converting subjective experiences into physiological changes (4). Anticipatory allostatic responses are induced in living organisms to aid in coping with and promoting survival when encountering real or perceived stressors. Over time, the ensuing allostatic reactions to these stressors can become more prolonged, either overly or underactivated. As a result, the typical regulatory set points and dynamic range change, resulting in the transition of transient allostatic reactions to allostatic states. AL is the result of prolonged cellular and physiological recalibrations caused by persistent activation of allostatic states. These include elevated levels of circulating stress mediators, hyperglycemia, and elevated blood lipid levels. AL, when maintained over time, sets off compensatory structural and functional recalibrations that lead to gradual dysregulation of the organism’s physiological network. The downregulation of hormone receptors on target tissues to prevent overstimulation and anatomical and circuitry remodeling of the brain in response to neurochemical variables are well-known physiological examples (4, 5). AL can also be caused by health-damaging behaviors, such as excessive alcohol consumption, smoking, drug use, poor dietary habits, lack of exercise, and irregular sleep patterns. These behaviors can worsen pathophysiological conditions by disrupting crucial biological mechanisms, such as inflammatory processes, subsequently intensifying AL (2). In this context, it is known that physical reactions to stress can be modified by the cognitive, emotional, and behavioral responses of an individual to particular stressors (6, 7).

Allostatic overload is a condition that occurs when a current source of distress, such as a recent life event or chronic stress, is identified and the stressor is determined to test or exceed the individual’s coping capabilities. Chronic exposure to persistent stressors, incapacity to adjust to repetitive stressors, incapacity to stop the stress response when a stressor is stopped and an insufficient allostatic response are situations that could result in triggering allostatic overload (8). Furthermore, allostatic overload causes molecular alterations at the cellular level, such as accelerated aging, which leads to the development and progression of diseases, premature morbidity and aging (4). Allostatic overload is associated with multiple symptoms that may cause a notable impairment in one’s ability to perform in social or professional settings, or both (8).

Transdisciplinary health studies have successfully employed the AL model to elucidate the link between chronic stress and diseases associated with high rates of disability and death, such as non-alcoholic fatty liver disease, chronic kidney disease, cancer, cardiovascular disease (CVD), and autoimmune and neurodegenerative disorders (9–15). For, example, it is well known that biological manifestations of allostatic overloading involve the immune system reaction dysregulation and promotion of chronic systemic inflammation, which has been recognized as a core mechanism in chronic disease progression (16, 17). To explain the human stress-disease cascade, an energy model of AL (EMAL) has been developed. Stressors increase the energy expenditure associated with allostasis, which depletes the reserve part of the organism’s total energy budget. Health-sustaining growth, maintenance, and repair activities are impacted when this extra energy cost surpasses the reserve capacity. According to this theory, reducing stress and promoting health throughout life can be achieved by many interventions that enhance an organism’s energy efficiency, and the key types of interventions; that have been recognized so far include exercise, calorie restriction, and meditation (4).

The observed inter-individual differences in morbidity can, in great part, be explained by differences in stress reactions, which can be influenced by various intrinsic (age, heredity, sex, emotional and cognitive appraisal of stressors, differences in coping strategies, health-related status) and external (variations in types of stressors, duration of exposure to stressors, interpersonal relationships, and social support network) factors (18). In particular, for an individualized approach to preventing chronic diseases, knowledge should be increased on the differences between men and women, and on the influence of aging on associations between reactions to chronic stress and accumulation of physiological damage, measured by AL, for which there is a substantial gap in knowledge. In this term, health disparities between men and women can be explained by their differences in exposure to stressors and susceptibility to particular stressors, which are not influenced only by biological sex, but also by psychosocial gender roles. This fact implies the importance of distinguishing between the term “gender” (experience of being male or female) and the term “sex” (genetic and biological characteristics) (19, 20). This review aims to clarify on sex/gender and aging as determinants of AL, which we believe is a potent area for future research.

## 2 Searching strategy

This is a state-of-art narrative review. The incentive to write it stemmed from our earlier research, which revealed that men and women, depending on their age, may follow different disease pathways and trajectories of health-related outcomes (21–24). This starting knowledge position assisted us in developing section titles, which included parts on sex/gender differences in biomarkers, biological and psychological components of AL, and the role of aging in stress and AL processes. A literature search revealed that there is a complicated interplay between biological and psychosocial factors that contributes to the development of numerous chronic diseases and multimorbidity via AL. We also identified significant treatments for reducing AL and preventing or slowing the course of chronic illnesses. To find relevant sources in the literature, we employed exploratory methods (browsing, followed by the snowball technique, searching references, and citation-tracking databases). We used a variety of sources to contextualize the texts we encountered. Medline (Ovid), PubMed (National Library of Medicine), Scopus (Elsevier), and Google Scholar were used for browsing. We used two overlapping groups of search phrases, combining “stress,” “allostatic load,” “chronic disease,” and “multimorbidity” with either the terms “sex”‘or “gender,” or “age” or “aging.” We did not select papers systematically, but rather based on the importance of the material for the intended sections. The inclusion criteria were systematic review publications and large population-based research, and we attempted to include as many recent studies as possible, with a goal period of 2017 to the middle of 2024.

## 3 Biomarkers of allostatic load – differences between men and women

The assessment of AL contributes to our understanding of lifestyle medicine; however, for a better understanding of AL contribution, it is recommended to utilize an integrated strategy that considers both biological indicators and clinimetric criteria. Biomarkers of AL are objective measurements of physiological reactions, while clinimetrics integrate these measurements with patient-reported symptoms and other subjective data (2, 25). Primary and secondary biomarkers crucial for determining AL have been identified in numerous studies. Primary mediators are markers of biochemical changes that occur in the neuroendocrine system at the onset of the stress response (26). These mediators, cortisol, epinephrine, norepinephrine, and dehydroepiandrosterone (DHEA), are linked to the stress response through the sympathetic-adrenal medullary axis and hypothalamic–pituitary–adrenal (HPA) axis. Secondary mediators are associated with the remodeling of receptor sites, resulting from prolonged activation of the stress response in the immunological, metabolic, and cardiovascular systems (26). Cardiovascular biomarkers, such as systolic and diastolic blood pressure, are commonly used as secondary indicators, while body mass index (BMI), waist-to-hip ratio (WHR), total cholesterol, high-density lipoprotein (HDL) cholesterol, and glycosylated hemoglobin are frequently used indicators related to metabolism (26). In comparison with individual biomarkers, the allostatic load index (ALI) was found to be a more accurate predictor of mortality and deterioration in physical functioning, including other biomarkers that also contribute to the AL response, such as C-reactive protein (CRP), interleukin-6 (IL-6), fibrinogen, pulse pressure, and apolipoprotein A1 (2, 27). Although biomarkers have contributed to a more accurate measurement of AL, they still fail to fully clarify this (9). Clinimetrics and psychosomatic studies have significantly contributed to the understanding and development of this field. In 2010, clinimetric criteria for identifying allostatic overload were introduced. Furthermore, in 2017, a modified version of a semi-structured interview accompanied by the Psycho-Social Index (PSI) was released and incorporated into the Diagnostic Criteria for Psychosomatic Research (DCPR) (2, 18, 28).

Each biomarker’s average levels differ markedly depending on the sex at birth; men have higher levels of cardiometabolic biomarkers, while women have higher levels of inflammatory and neuroendocrine biomarkers, which may explain why men are more likely to suffer from metabolic disorders and women with autoimmune diseases (29, 30). In laboratory testing, men elicit higher levels of cortisol and ACTH than women in response to acute psychosocial stress (29). Neuroendocrine markers (epinephrine and norepinephrine) and certain markers of the immune system (IL-6, CRP, and fibrinogen) in men have been identified as markers that correlate well with high AL. Therefore, elevated values are considered high-risk factors for the occurrence of allostatic overload (28, 29). Among women, high-risk clustering also includes CRP, IL-6, glycated hemoglobin, and systolic blood pressure (28, 29). Owing to the significant sex-related variation in individual biomarkers, it is recommended to establish specific biomarker cut-off values for each sex (gender) when assessing their impact on AL (30).

## 4 The biological components of allostatic load – differences between men and women

The brain is the main organ responsible for processing all inputs associated with significant life events and environmental stress. The response to stress involves the coordinated activity of the autonomic nervous system (ANS), HPA axis, and neural circuits of the cortical–limbic brain regions that are known to mediate higher-order cognition and emotion regulation, including the prefrontal cortex, ventral striatum, amygdala, and hippocampus (22). The cardiovascular, immune, and metabolic systems also play roles in this response. This highlights the importance of using a range of biomarkers to evaluate AL (2).

However, the neuroendocrine system, which responds to stimuli by activating the HPA axis, is considered to be a key player in restoring homeostasis. Therefore, sex variations in HPA and behavioral responses to stress could be the key mechanisms to explain the observed sex biases in the risk of stress-related diseases (31). Systematic reviews showed that compared to women, men seem to have a larger AL overall, with the caution that sociocultural gender-based variables may influence within-sex differences in stress response patterns (29). In laboratory testing, men elicit higher levels of cortisol and ACTH in response to acute psychosocial stress than women. Experiments have indicated that progesterone is negatively associated with the ACTH and cortisol responses in women (32).

The greater prevalence of depression in women can be explained by the specific reproductive events marked by fluctuations in estrogen levels; however, recent studies indicate that androgens also play an important role in regulating the HPA axis, modulating it directly by androgen receptors or by affecting the estrogen signaling pathway (33, 34). Women are more susceptible to autoimmune diseases, which are also associated with HPA axis hyperactivity. Conversely, men are more likely to suffer from conditions such as diabetes mellitus (DM) and CVD, which are characterized by high AL and permanent and excessive activation of the stress system (32).

In addition to differences in HPA axis activity, studies have demonstrated that men and women activate different brain networks involved in stress responses, which enables them to elicit effective coping mechanisms. Men’s prefrontal cortex and women’s limbic/striatal regions show higher stress responses, according to a study that used functional neuroimaging (fMRI) to examine sex differences in neural responses during stress (35). Differences can also be observed in the expression and signaling of corticotropin releasing factor (CRF) receptors and in the regulation and production of CRF by neurons. The observed differences may predispose women to be more responsive to stress and more prone to the development of disorders characterized by CRF dysregulation, including post-traumatic stress disorder (PTSD), panic disorder, and major depression disorder (31).

## 5 Psychosocial factors influencing differences between men and women in reaction to chronic stress

### 5.1 Gender related differences in reaction to stress

Women show a higher life expectancy than men but a higher morbidity burden (the female–male health-survival paradox) (19). This can be partly explained by gender roles (male–female) and traits (masculinity-femininity). Owing to their social roles, women are usually more exposed to stressful situations than men. For example, women are more likely to be caregivers. They are more exposed to conditions of burden, such as longer hours of caregiving, less help from others, relational and financial problems, and problems combining different tasks (36). In addition, they differ from men in susceptibility to stress (the strength of the effects of stressful situations on their physiological burden and AL), mainly through differences in cognitive appraisal of stressors and coping (emotional, cognitive, and behavioral responses to stressful situations) (19).

The degree to which people adopt stereotypically masculine or feminine behaviors is a good indicator of their gender roles, which are frequently influenced by how they perceive themselves as male or female, respectively. Notably, independent of biological sex, characteristics linked to masculinity predicted a higher AL. In professions that are, for example, predominantly male, women are more likely to develop higher AL than men because they take on the characteristics of the male milieu to which they are associated, and at the same time, by adapting, they try to gain the respect of their male colleagues, which requires additional effort and leads to stress (37). In the framework of stress reactivity research, several studies have found that sociocultural gender influences cortisol dynamics, which was previously thought to be predominantly defined by biological sex. The findings revealed that sexual orientation modulates free cortisol dynamics in distinct gender-based patterns, with lesbian/bisexual women exhibiting peak cortisol concentrations late during recovery from a stressor (40 min after exposure) compared to heterosexual women (peak cortisol at 10–20 min after exposure). In contrast to the findings for women, gay/bisexual males had lower total cortisol concentrations during testing than heterosexual males. These findings show that gay/bisexual males may downregulate the HPA axis, although lower cortisol levels may also signal the development of adaptive coping techniques to protect from the stress response and AL (38).

### 5.2 Sociodemographic and lifestyle determinants of allostatic load and differences between men and women

Many sociodemographic factors, such as age, sex, gender, socioeconomic position (SEP), education level, and lifestyle (engaging in physical activities, alcohol consumption, smoking, and substance addiction), influence an individual’s reaction to stress and contribute to gender-related disparities in health (39). Higher SEP has been proven to be related to lower AL in both men and women in several trials (41–43). Parental SEP has an inverse relationship with midlife AL in both sexes, with education acting as a partial mediating factor in this relationship (40). Longer periods of poverty during childhood are associated with higher AL trajectories from childhood to adulthood. For example, a study of Swedish women with lower SEP in childhood revealed that they had greater adult AL, supporting the link between high AL and low SEP (41, 42).

Differences in gender-related roles and SEP make men and women distinctly exposed to stressors, for which reason they may differ in their AL scores. The recent literature involves two other concepts that help explain health inequalities among people in the population. These are concepts of susceptibility (when the effect of similar stressors on AL differs across groups) and vulnerability differences among groups in the availability of resources to cope with stressors (43).

These three mechanisms: differential exposure to stress (unequal distribution of some stressors across the groups), susceptibility, and vulnerability to similar stressors, are not mutually exclusive. Recent improvements in methodology to estimate interactions and mediation effects between different sociodemographic factors are especially helpful in understanding the role of socioeconomic inequalities in health (how an individual’s social position influences disease risk). This possibility of equalizing the exposure or effects of stress across social groups allows us to set targets and priorities when planning health policies. The inclusion of these composite measures of the reaction to stress in population studies also helps to reveal gender-related inequities in health. For example, in a large pan-European study, it was investigated whether socially disadvantaged individuals were more susceptible to the detrimental effects of smoking and alcohol intake on AL (44). The results showed a larger effect of these risk factors on AL in low-educated men than in low-educated women, even after justification of exposure to these risk factors. Differential exposure and susceptibility mechanisms of sociodemographic factors were also shown to be relevant in understanding the pathway from risk factors, through AL, to disease onset (45).

Early adversity is also thought to be correlated with AL, and women appear to have stronger connections between AL and early life issues. In particular, abuse and neglect during childhood have been associated with elevated AL in middle-aged women. Women who reported childhood sexual assault had elevated levels of hair cortisol, total cholesterol, triglycerides, and low-density lipoprotein (LDL) cholesterol, all of which contribute to allostatic overload (46, 47).

The impact of marital status on health and wellness was also demonstrated. Notably, it seems that women’s health is more strongly affected by these relationships. In particular, women’s AL is lower when they are married or cohabitating (29, 48).

Research has linked AL to several workplace factors, including insufficient recuperation from occupational stress, changes in job requirements and organizational structure, and mismatch between effort and compensation. These issues are particularly prevalent among female workers and are considered the main contributors to the development of burnout syndrome (2, 49). The association between AL and gender differences in workplace stress has been acknowledged, but relationships are unreliable and often overlook home and family factors that may intensify gender-specific stress (29, 50). A large population study in the United Kingdom investigated the impact of work schedule on the presence of AL biomarkers of chronic stress. The research results indicated that the ability to control work schedules was linked to lower AL among women but not men. Women who adhered to traditional gender roles experienced the greatest reduction in AL when given the opportunity to regulate their work schedules (51). High work expectations, along with insufficient control at work, have significantly increased employees’ cardiovascular risk in both men and women (52). Work-related stress scenarios were found to co-occur with unhealthy lifestyle choices, such as excessive alcohol consumption, smoking, low levels of physical exercise, and obesity, managing to raise AL (53).

Education reduces the incidence of AL in older age, supporting preventive strategies based on educational achievement to enhance older adults’ health (54). Research shows AL levels are similar across races/ethnicities among those with little education, but the greatest AL differences occur among college-educated individuals. These findings suggest that socioeconomic inequalities by race/ethnicity result from uneven educational returns, increasing stress among minorities (55).

The architectural environment also significantly impacts allostatic overload. Analyzing stress-inducing architecture suggests long-term exposure to such forms may worsen allostatic overload, potentially leading to systemic inflammation. Increased urbanization and extended indoor periods likely exacerbate this issue for both genders (56, 57).

### 5.3 Coping mechanisms differences between men and women

The behavioral strategies an individual will choose to cope with a stressful situation depend on many factors, such as age, health status, internal psychological resources, personality type, education, previous experiences, financial assets, and social support (58). The two main types of coping strategy are emotion-focused and problem-focused. Problem-focused coping is characterized by behavioral and cognitive efforts to change or abolish stressors. In contrast, emotion-focused coping, which is typically thought to be less effective than problem-focused coping, aims to alter emotional responses to stressor (59).

It is believed that sex influences coping mechanisms, as men and women are socialized to handle stress differently (60). Men generally use problem-focused coping techniques more, while women prefer emotional coping methods. Problem-focused coping, associated with better health outcomes, aligns with masculine traits. Femininity shows mixed associations with active coping (61). Women employing emotion-focused coping face higher rates of depression and anxiety, though study results vary (59). Women report more stress and higher scores in daily and chronic stress due to their emotional coping and avoidance methods, unlike men who typically exhibit more emotional restraint (62). During the COVID outbreak, emotion-focused coping showed benefits; men’s tendency for active coping may have increased their anxiety, whereas women’s preference for emotion-focused approaches and positive reframing may have protected their mental well-being (63).

## 6 The sex/gender –related risk factors interplay in chronic diseases connected to chronic stress reactions

The response to chronic stress develops as a complex interaction of biological and psychosocial (gender-related) factors, including social roles and coping mechanisms, which leads to changes in the body. These changes have been linked to many chronic diseases in modern society through AL (2, 64). A higher AL was also found to be a predisposing factor for the development of common community diseases and a consequence of the disease burden. However, this is difficult to clarify in cross-sectional studies. In general, current study designs rarely consider the interaction effects of different risk factors or demonstrate the risk factor cascades or networks (65). A higher AL was found in prospective studies to increase the risk for CVD, playing the role of an intermediate factor in the effect of a risk factor burden on disease onset (ref. 45, 66). Many studies found associations of higher AL with different entities of CVD, such as peripheral arterial disease (PAD), ischemic heart disease (IHD), coronary arterial disease (CAD), and atrial fibrillation (AF) (67, 68). Up to a third of patients with AH and congestive heart failure (HF) were shown to have elevated AL (69, 70). Higher AL was found to be associated with a higher overall and CV mortality (71).

Stress-related disorders (SRDs) with cortisol blunting (inability to exhibit a normal increase and fall of cortisol in response to stress) are more prominent in women. Cortisol blunting, an indicator of SRDs, is considered to be associated with both gender, as a psychosocial variable, and sex as a biological variable (72). Therefore, the association between sex/gender and CVD may be significantly influenced by stress and AL. While biological risk factors and unhealthy behaviors may cause higher AL in men, chronic stress and psychosocial variables may better explain the patterns of increased AL observed in women. In fact, women exhibit more dysregulation in neuroendocrine and immunological functioning, while men exhibit AL patterns that are more closely linked to compromised anthropometric, metabolic, and cardiovascular performance. Therefore, it is possible that gender-related characteristics, particularly through stress processes, contribute to the etiology of CVD (73).

With respect to CVD, there are some physiological benefits associated with the female sex, but they appear to vanish as soon as women lose the protective effect of estrogen in the postmenopausal period (21). Men and women follow different paths in the aging of blood vessels, and both sex-and gender-related factors play a role in creating these differences. For premenopausal women, one of the most crucial sex-related factors in preventing blood vessel aging is the natural 17ß-estradiol level. Women experience a sharper increase in the rate of blood vessel aging than men, but gender-related factors are also significant. Women tend to experience psychological stress, depression, particular psychological traits, and lower SEP more often than men do, and these conditions are expected to have a greater impact on blood vessel aging in women. Conversely, men are more vulnerable to the negative effects of alcohol use and social deprivation on blood vessels (74).

AH is an important modifiable risk factor for CVD. According to several observational studies, women have a higher correlation between blood pressure and the risk of CVD (75). Identifying significant contributors to hypertension-related cardiovascular outcomes can also be achieved by using sex-and gender-based perspectives. In men, incident hypertension starts to increase after adolescence and steadily increases with age. In women, the critical period when hypertension starts to emerge is perimenopause, which coincides with estrogen’s drop-down, and an emergency of abdominal-type obesity and metabolic and inflammatory diseases (23). The molecular basis of oxidative stress, inflammation, dysregulation of the renin-angiotensin-aldosterone system (RAAS), and genetic predisposition also appear to account for the sex disparities in AH (76). Studies have shown that innate and adaptive immune responses are regulated differently by sex, potentially leading to sex-dependent vascular inflammation and AH development (21, 77). In addition, women with AH are found to be more affected than men by factors such as life stresses, workplace-related anxiety, and depression. Both unmarried women and married men are less likely to have AH (78). Currently, studies on the effects of dietary salt intake on sex-specific renal processes are ongoing. Intervention with dietary salt leads to a higher release of the cytokine tumor necrosis factor-*α* (TNF-α) in women, which may hinder the activity of NaK2Cl cotransporter type 2 (NKCC2) and enhance the salt-dependent increase in blood pressure. Sex hormones, gender, and sex-specific molecular pathways influence the metabolism of glucose and lipids, as well as the energy metabolism and heart function (e.g., in women, there is a significant increase in the use of myocardial fatty acids during exercise, a decrease in cardiac fibroblast collagen synthesis due to estradiol, and a weak downregulation of mitochondrial genes in HF) (79).

In addition to CVD, increased AL plays an important role in patients with DM (80). Higher AL was found to be linked to more brain amyloid build-up, which implies that it might be involved in the pathophysiology of Alzheimer’s disease (AD) and dementia (81). In addition, higher levels of AL were found to be linked to poorer spine bone mineral density and fibromyalgia in women in a cross-sectional investigation. Women with breast and ovarian cancer have higher basal cortisol levels and lower acute cortisol reactivity than healthy controls and cancer survivors, in both men and women, and are more likely to experience allostatic overload (82–84). However, the risk of cancer death was found to be highest in obese patients with high AL, which confirms the importance of behavioral risk factors in the impact of AL on overall health (85).

The significance of the impact of AL on the emergence of diseases has been particularly highlighted in relation to the development of psychological disorders and episodic or chronic migraines. Several studies revealed a strong correlation between AL and depression or anxiety, and AL seemed to play a mediating role in the relationship between physical assault during childhood and depressive disorders in adulthood (2, 86, 87). However, high AL was more strongly linked to depression in women than in men, which could be connected to emotion-focused coping mechanisms mostly used by women (88). In patients with psychosis, high AL seemed to be negatively correlated with psychosocial and cognitive functioning (89). These findings support the idea that mental disorders should be reinterpreted as systemic diseases that affect the brain and other biological processes, leading to systemic comorbidities. All the comorbidities discovered in relation to metabolism, immune, psychological and cardiovascular system might be the result of long-term damage caused by the complex interplay of risky health behaviors and allostatic overload depending on sex/gender characteristics. Knowing the complexity of the occurrence of these disorders and including the importance of allostatic overload in their pathophysiology, we could predict the onset of physical chronic diseases in people with mental disorders and include early interventions aimed at reducing AL and/or improving coping mechanisms to prevent deterioration (90).

## 7 Age perspective of the reaction to chronic stress and allostatic load

Aging is considered a major risk factor for the development of common chronic diseases, including CVD, cancer, and neurodegenerative diseases, because of the accumulation of damage in cells and tissues and the lowering of the fitness of the body (91). Although chronic diseases and functional deficits are stressful, recent studies have shown that levels of psychological resilience (the ability to maintain psychological stability and well-being despite experiencing adversities) may be higher in the elderly than in their younger counterparts (92). These characteristics of older people are associated with years of experience and learning stress management skills (93). In the context of a decline in physical functions with aging, higher levels of psychological resilience in this age may be a reason for maintaining the AL score relatively stable (94).

Different trajectories were suggested for men and women based on the positive and significant coefficients for age. AL trajectories based on sex show a clear female advantage, aligning with the female advantage in life expectancy (95). Higher baseline AL scores were found to be associated with a significantly higher risk of 7-year mortality and decline in both mental and physical abilities. A marginal association with CVD events was also observed. These findings remained consistent, even after accounting for standard sociodemographic characteristics and baseline health status (96).

Aging typically involves a gradual decrease in physiological and psychological variance, which may make traditional AL biomarkers less applicable to older populations because of reduced systemic variance over time. Therefore, it is important to consider which biomarkers should be used to assess AL in the elderly (97). For instance, dopamine indicators decrease by 40–50% between the ages of 18 and 88, and aldosterone concentrations may decrease by up to 50% by the age of 70 (98). In older individuals, allostatic biomarkers can be categorized into three groups: those that become less variable with age (e.g., aldosterone), those that retain significant variance to show an adaptive stress response (e.g., BMI and immune markers), and those that exhibit variability and reactivity in response to immediate stressors (e.g., cortisol, epinephrine, and creatinine), representing sustained systemic variability (99). Allostatic biomarkers that are significantly variable in the elderly, such as cortisol-DHEA ratio, epinephrine, norepinephrine, IL-6, CRP, fibrinogen, HDL-cholesterol, creatinine, and systolic and diastolic blood pressure, should be used to identify reactions to external stress. Studies have identified BMI and IL-6 as two parameters that are mostly indicative of worsening health in older, generally healthy individuals (100).

## 8 Can we use the knowledge about sex/gender and age differences in allostatic load for better understanding of multimorbidity?

Multimorbidity, sometimes referred to as multiple long-term diseases (MLTC), is the coexistence of two or more chronic illnesses in one individual. MLCT is associated with increased rates of early death, significant reductions in functioning and quality of life, and increased usage and cost of health and social care. The growing burden of MLTC makes it imperative to understand the risk factors that may prevent its accumulation. In the United Kingdom, 33% of patients receiving primary care in 2019 had three or more MLCT, and approximately 50% had two or more MLCT. The prevalence increased by over 70% between 2004 and 2019, and a greater increase is expected as the population ages (101). Multimorbidity increases the risk of both physical and mental impairment, and it is becoming a critical issue for health systems worldwide as the population of adults over 65 years of age continues to grow at an unprecedented rate and is expected to reach over 1.5 billion by 2050. A growing amount of research is being conducted to determine the causes of multimorbidity. AL and frailty (reduced homeostatic reserves in multiple organs and systems) appear to be common factors in many causes. In our day-to-day clinical work, we witness that many clinical recommendations for risk assessment and therapy are not successful in patients with multimorbidity; therefore, innovative approaches to multimorbidity are desirable (24).

Numerous studies have identified both direct and indirect links between AL and multimorbidity development. A longitudinal American study revealed a significant association between AL and multimorbidity progression, even after accounting for socioeconomic and behavioral factors. This study suggested that AL, driven by adverse socioeconomic conditions, mediates the development of multimorbidity (102). Additionally, research involving nearly 40,000 participants found a graded relationship between biological disturbances, multimorbidity, and self-reported childhood problems. Approximately 80% of those with very difficult childhoods had multimorbidity compared to 44% of those who perceived their childhood as very good. Differences in childhood experiences were more commonly reported among women, individuals with lower education, less physical activity, and those with sleep disorders (103). Adverse childhood experiences have been linked to adult multimorbidity, though evidence for the influence of biological and psychological factors is limited. A Canadian Longitudinal Study of Aging introduced a mediation model showing that adverse childhood experiences were directly and indirectly associated with multimorbidities across all age cohorts for both males and females (104).

The adaptive mechanisms of the CNS, HPA axis, immunological system, and metabolic system underlie psychological resilience. Excessive activation of these systems can trigger pathophysiological events in other organs, increasing the risk of chronic illnesses. Long-term psychological stress is known to accelerate aging by depleting homeostatic reserves and through overlapping physiological, cellular, and molecular pathways. However, the cellular and molecular processes by which chronic stress contributes to chronic illness development are not fully understood (22). Geroscience argues that the rise in age-related disease susceptibility and disability is primarily due to the biological processes of aging. Significant correlations have been found between various health factors and age-related traits, particularly in autophagy, mitochondrial function, cellular aging, and DNA methylation. Activating and managing these resilience mechanisms in well-aged individuals could lead to groundbreaking medical discoveries. Currently, a variety of classification techniques are used to better understand multimorbidities. Among the most often used is cluster analysis, which looks at which specific illnesses are more likely to co-occur (or cluster) in multimorbidities (105).

Potential explanations for the association between multimorbidity and allostatic overload include oxidative stress, metabolic dysfunction, dysregulation of the HPA axis and ANS, accelerated aging, and telomere shortening. Because allostatic overload impairs immunological function and decreases self-management abilities, it may worsen preexisting multimorbidity. A better understanding of sex/gender and age-specific differences in AL can facilitate the development of targeted interventions to reduce allostatic overload and enhance outcomes in multimorbidity. These interventions include stress management techniques, lifestyle modifications, social support, community engagement programs, and pharmacological approaches to modulate stress response systems (105). Research has demonstrated that allostatic AL mechanisms influence not only the initiation of long-term illnesses but also the aging process and the development of multiple concurrent conditions. Consequently, gaining deeper insights into how AL specifically relates to sex, gender, and age could prove invaluable in identifying biomarkers for patient categorization. Moreover, future studies should investigate the relationship between the established AL biomarkers and all other environmental factors that contribute to these variations.

## 9 Preventive measures that can alleviate allostatic load

Researchers are examining psychological well-being’s role in maintaining allostasis to understand the link between wellness, well-being, and allostasis. Studies consistently show connections between emotional well-being, life harmony, and psychological wellness (106). Notably, women with a positive emotional profile tend to have a favorable AL profile, indicating that positive emotions may help prevent various diseases and multimorbidities (107).

The link between AL and well-being has increased interest in enhancing well-being through nutrition control, sleep management, physical exercise, and relaxation techniques like meditation. These practices can reduce AL, improving stress response and reducing discomfort, which helps prevent chronic diseases, especially multimorbidity influenced by allostatic overload. Studies, for example showed that a healthier diet positively impacts lowering AL in individuals over 30 and those with metabolic disorders (108, 109).

Exercise reduces pro-inflammatory molecules and boosts anti-inflammatory cytokines, thereby lowering overall inflammation and potentially strengthening the immune system, which may reduce chronic diseases, particularly those with immune backgrounds (110). It also positively affects brain function and gut microbiota. In the brain, exercise enhances sleep, mood, memory, cognitive flexibility, and reduces depression. Indirect brain benefits arise from modifying gut microbiome diversity, improving gut motility and increasing of antioxidant enzymes, and anti-inflammatory cytokines (111). Sedentary behavior is associated with higher ALI, while physical exercise mitigates AL. It is therefore possible that physical inactivity directly impacts AL, harming gut microbiota and brain health, increasing susceptibility to these diseases (112).

Sleep deprivation increases the body’s response to stress, making it more susceptible to the negative effects of stress, potentially creating a harmful cycle, and contributing to the development of AL due to various risk factors, including poor lifestyle choices and low SEP (113). A strong negative link between sleep disturbance and increased AL was found, with less correlation in samples that included a higher percentage of female participants (114).

Studies on women’s mental health reveal why employed women suffer poor mental health despite contributing to household income in family-friendly work environments. Findings show job autonomy improves mental health, but benefits depend on spousal gender views. Women with partners holding egalitarian gender ideologies reported good mental health regardless of job autonomy, whereas women’s own gender ideologies had little predictive value. Thus, altering men’s gender ideologies at the societal level can be crucial for enhancing employed women’s family well-being and reducing chronic stress and AL (115).

Considering everything written, improving AL requires a multifaceted approach that includes healthy eating, regular physical activity, mindfulness techniques, gender ideology changes, and sleep hygiene maintenance. These methods have generally been successful across sexes, but should be selected based on stress and AL patterns specific to each sex/gender (73). Moreover, successful aging today focuses on high psychological, social, and physical resilience rather than the absence of chronic diseases. Hence, preventive measures for older adults should enhance resilience, considered vital for successful aging. These include problem-solving coping styles, positive emotions (optimism, hopefulness, life satisfaction), community involvement (social roles), and improving mobility and perceived health (22).

## 10 Conclusion

The increase in the prevalence of chronic diseases, primarily cardiovascular, metabolic, immunological, malignant, and psychiatric diseases, combined in multimorbidity, is based on a modern lifestyle. Due to unfavorable environmental factors, an accelerated pace of life, changes in food production and processing, changes in sleep patterns and habits, basic human ritual changes, and negative behavior patterns are also adopted. All these factors lead to obesity, increased levels of inflammation and other important changes that form the pathophysiological basis of chronic diseases, and multimorbidity (116). Awareness of the impact of acute and chronic stress, as well as AL, on the development of these diseases has increased over the past decade. Many studies have confirmed that AL, evaluated using biomarkers and ALI, can serve as a helpful indicator of general health and a method to understand the underlying causes of aging (117). AL is linked to various health conditions, making it an indication of biological and physiological stress (118).

**Figure 1:**
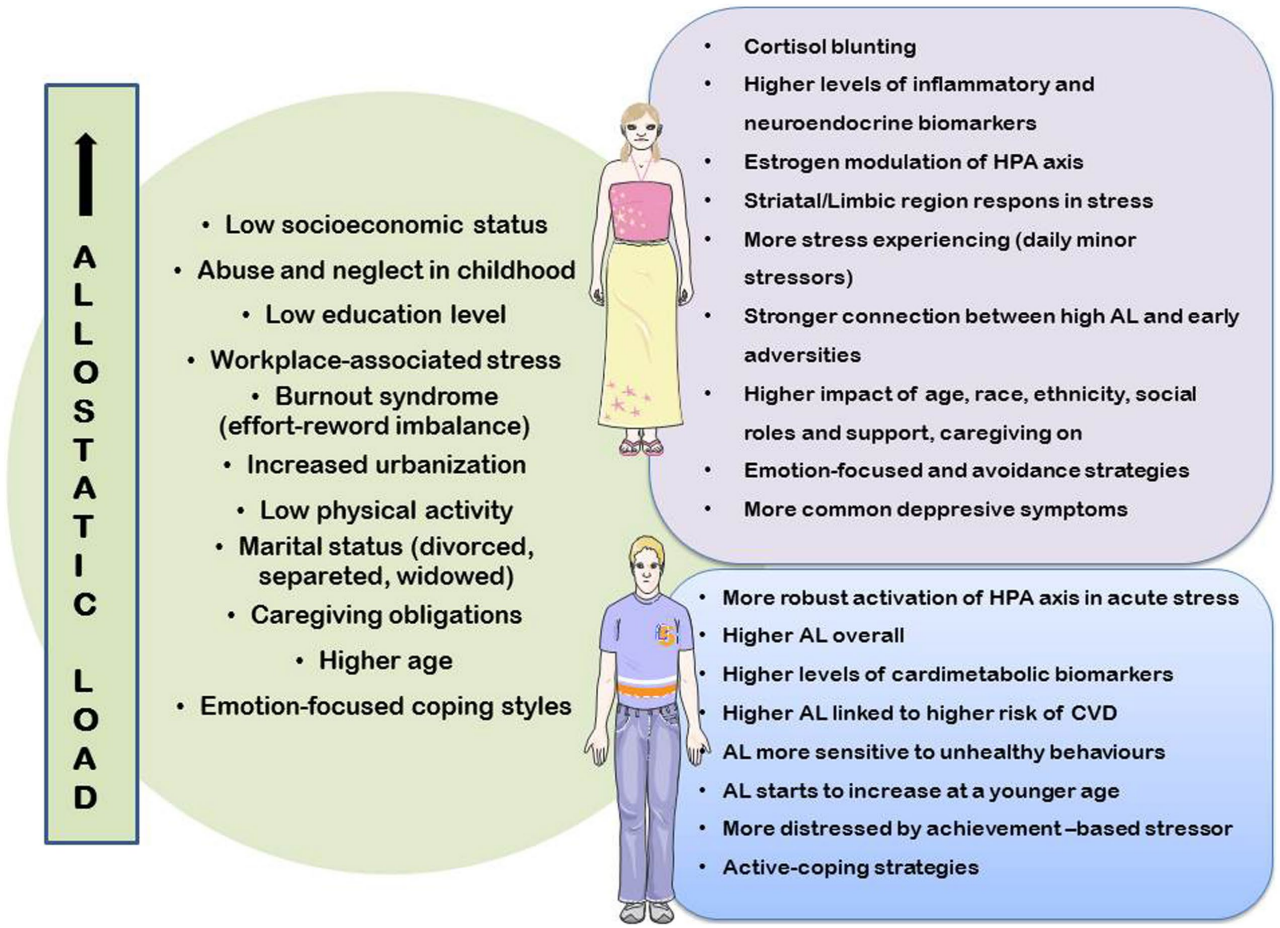
Factors related to allostatic load generally and depending on sex/gender differences.

This paper synthesizes the available information on AL differences depending on sex, gender, and age, and provides a model for the study of AL, with a focus on these differences. This model can be incredibly valuable not only for gaining a better understanding of the differences in the frequency of incidence of different diseases based on sex, gender and age, but also for developing preventive strategies based on the aforementioned disparities to prevent chronic diseases (Figure 1). Currently, many studies do not take these AL differences into account when analyzing these methods. This may be due to the fact that tools for measuring AL are still poorly employed in clinical settings, with cut-off values seldom adjusted for sex and age.

There is a need to change clinical practice, to shift from curative to preventive medicine and health promotion, especially in terms of the need for systematic AL profiling and the application of preventive measures such as raising psychological resources and learning harmless stress response strategies. This suggests a need for more extensive studies on the psychosocial elements that negatively impact the body’s stress response and AL. Such research should focus on how these factors interact and their varying effects across different social groups, with particular emphasis on gender differences and older populations.

To support changing practice routines, intervention trials that examine the efficiency of stress reduction approaches on the development of multimorbidity and health-related outcomes will be required, e.g., studies investigating the genetic and epigenetic variables that determine vulnerability to AL and multimorbidity. Such research would provide us with sufficient knowledge to develop personalized approaches to managing AL and prevent multimorbidity, such as tailored stress management interventions based on sex, age, and cultural factors; precision medicine strategies based on individual biomarker profiles; and the use of artificial intelligence to predict individual risk while accounting for sex/gender and age differences (100).

It is certainly necessary to empower the elderly population in terms of functional abilities and to train them to maintain their independence in everyday life for as long as possible. This transformation of the healthcare system will be long-term; however, awareness of the connection between psychosocial factors and AL with the onset of chronic diseases and functional deficits can help this transformation. In this sense, this work is expected to have a significant impact on future research and will hopefully change clinical practices.

To summarize, future research in this area has the potential to transform our approach to health management and illness prevention. By combining modern technology, longitudinal investigations, and multidisciplinary collaborations, we can get a better understanding of the complex mechanisms driving AL and multimorbidity, leading to more effective solutions for promoting health and well-being in various groups.
